# Abnormalities in Cortical GABAergic Interneurons of the Primary Motor Cortex Caused by *Lis1 (Pafah1b1)* Mutation Produce a Non-drastic Functional Phenotype

**DOI:** 10.3389/fcell.2022.769853

**Published:** 2022-03-02

**Authors:** E. Domínguez-Sala, L. Valdés-Sánchez, S. Canals, O. Reiner, A. Pombero, R. García-López, A. Estirado, D. Pastor, E. Geijo-Barrientos, S. Martínez

**Affiliations:** ^1^ Instituto de Neurociencias, Universidad Miguel Hernández—CSIC, Sant Joan d'Alacant, Spain; ^2^ Department of Molecular Genetics, Weizmann Institute of Science, Rehovot, Israel; ^3^ Centro de Investigación Biomédica en Red en Salud Mental CIBERSAM, Madrid, Spain

**Keywords:** fast-spiking interneuron, cortical inhibition, oscillatory activity, motor cortex, mental disorders

## Abstract

*LIS1 (PAFAH1B1)* plays a major role in the developing cerebral cortex, and haploinsufficient mutations cause human lissencephaly type 1. We have studied morphological and functional properties of the cerebral cortex of mutant mice harboring a deletion in the first exon of the mouse *Lis1* (*Pafah1b1*) gene, which encodes for the LisH domain. The *Lis1/sLis1* animals had an overall unaltered cortical structure but showed an abnormal distribution of cortical GABAergic interneurons (those expressing calbindin, calretinin, or parvalbumin), which mainly accumulated in the deep neocortical layers. Interestingly, the study of the oscillatory activity revealed an apparent inability of the cortical circuits to produce correct activity patterns. Moreover, the fast spiking (FS) inhibitory GABAergic interneurons exhibited several abnormalities regarding the size of the action potentials, the threshold for spike firing, the time course of the action potential after-hyperpolarization (AHP), the firing frequency, and the frequency and peak amplitude of spontaneous excitatory postsynaptic currents (sEPSC’s). These morphological and functional alterations in the cortical inhibitory system characterize the *Lis1/sLis1* mouse as a model of mild lissencephaly, showing a phenotype less drastic than the typical phenotype attributed to classical lissencephaly. Therefore, the results described in the present manuscript corroborate the idea that mutations in some regions of the *Lis1* gene can produce phenotypes more similar to those typically described in schizophrenic and autistic patients and animal models.

## Introduction

The functional properties of the cerebral cortex in the adult depend on the correct cortical development, which is a complex process that commences prenatally and lasts up to later postnatal stages. Among the many genes that are involved in cortical development, *LIS1* (HUGO gene nomenclature committee name: *PAFAH1B1* gene, platelet activating factor acetyl hydrolase 1b regulatory subunit 1) has a key role (Reiner et al., 1993). The product of this gene, the protein LIS1, has two major functions. First, it is a regulator of the cytoplasmic dynein molecular motor, which has crucial roles during axonal transport, nuclear migration, cell division, cell polarity, and microtubule dynamics; to carry out this function, LIS1 forms homodimers through the interaction of LisH domain from each polypeptide (reviewed by Reiner and Sapir 2013). Second, the LIS1 protein is the beta subunit of the platelet-activating factor acetyl-hydrolase (PAF-AH), an enzyme that regulates platelet-activating factor (PAF) levels (Hattori et al., 1994). PAF has several functions in the CNS, such as modulating excitatory synaptic transmission (Clark et al., 1992; Bazan 2003) or controlling neuronal branching stability (Gopal et al., 2009).

The *LIS1* gene was initially discovered as the gene responsible for Miller-Dieker’s syndrome (MDS), and soon afterwards, it was concluded that alterations in *LIS1* were sufficient to produce the isolated form of lissencephaly (lissencephaly type 1, LT1; Reiner et al., 1993; Nigro Lo et al., 1997). Its coding sequence is located in the so-called Lissencephaly Critical Region (LCR), where several essential genes for CNS development are encoded (such as *LIS1* or *YWHAE*), which shows a high mutation rate. In the case of *LIS1*, these mutations vary from punctual nucleotide modifications to long deletions (Chong et al., 1997; Tabarés-Seisdedos et al., 2006). The most characteristic symptoms associated with alterations in *LIS1* are severe cortical dysplasia (agyria or pachygyria) and frequent epileptic episodes, resulting in an extremely high mortality rate during early postnatal stages (Dobyns et al., 1993; Reiner et al., 1993).

The studies carried out using animal models of *LIS1* dysfunction have been mainly focused on the impact of modified *LIS1* expression during embryonic and perinatal brain development. The mouse gene *Lis1* (*Pafah1b1*) is the homologous of the human *LIS1* gene and participates in cell proliferation in the developing brain (Hirotsune et al., 1998; Cahana et al., 2001; Shu et al., 2004; Bi et al., 2009; reviewed in; Reiner and Sapir, 2013). Studies using murine genetic models with mutations in the *Lis1* gene showed that both increase and decrease in *Lis1* expression result in a disorganization of neural precursors in the ventricular layer and an abnormal cell migration of neuroblasts in the cerebral cortex (Hirotsune et al., 1998; Cahana et al., 2001; Gambello et al., 2003; Bi et al., 2009; Pramparo et al., 2011), as well as abnormal development of the cerebral cortex (Cahana et al., 2001) and subcortical structures (García-López et al., 2015).

Several studies have analyzed the consequences of *Lis1* mutations on brain physiology or morphology in postnatal stages. It has been shown the presence of excitatory and inhibitory synaptic abnormalities in the hippocampus (Jones and Baraban, 2007, 2009; Wang and Baraban, 2008; Greenwood et al., 2009; Hunt et al., 2012; Sudarov et al., 2018) and cortex (Valdés-Sánchez et al., 2007; Sudarov et al., 2013). In addition to postnatal synaptic abnormalities, a mouse model presenting a deletion in the LisH domain from one allele showed a decrease in the number of some GABAergic interneuron subtypes in the anterior cingulate area and abnormalities in the septo-hippocampal cholinergic projection (García-López et al., 2015; 2021). However, there is still a lack of knowledge regarding the pathophysiology of *LIS1* dysfunction and the relationship between alterations in different portions of the LIS1 protein and their structural and functional consequences in the postnatal brain (genotype-phenotype relationship). The possibility that different *LIS1* alterations may produce different pathologies is highly relevant since some authors have proposed that alterations in some portions of the gene encoding for LIS1 may be implicated in neurological disorders less severe than lissencephaly, such as schizophrenia, autism, and bipolar disorder (Reiner et al., 2006; Tabares-Seisdedos et al., 2006; Tabares-Seisdedos et al., 2008). In a recent work performed using a mouse lacking the LisH domain from one allele (the *Lis1/sLis1* mouse) we reported some structural alterations compatible with the ones described in schizophrenic patients (García-López et al., 2021): a reduction in the number of GABAergic interneurons in the anterior cingulate cortex, a disbalance in cellular activation as assessed by c-fos expression, and alteration of behavioral tests related to these cellular deficiencies. These abnormalities are consistent with findings in schizophrenic patients (Hasimoto et al., 2003, 2008; Fung et al., 2014; Glausier et al., 2014).

This work is aimed to determine the impact of a deletion of the LisH domain on the anatomical organization and electrophysiological properties of the anterior cortical portion, specifically in the primary motor cortex. We selected this cortical area to extend the findings of García-López et al. (2021) to a non-associative cortical area, the primary motor cortex. The primary motor cortex has the advantage that, in addition to structural methods, it can be studied with electrophysiological (functional) techniques, such as “*in vitro*” slice electrophysiology and field potential recordings in the intact brain; this would allow a more complete understanding of the alterations caused by the deletion of the LisH domain. The motor cortex has been implicated also in the origin of psychomotor abnormalities present in psychiatric disorders including schizophrenia as a consequence of the abnormal modulation of the motor cortex activity caused by misbalanced activity coming from subcortical structures (Northoff et al., 2021); therefore, a detailed understanding of the alterations of the primary motor cortex caused by *Lis1* dysfunction could contribute to clarify the pathophysiology of lissencephaly and other brain diseases.

## Materials and Methods

### Ethics Approval

Mice were maintained, handled, and sacrificed in accordance with national and international laws and policies (Spanish Directive “Real Decreto 1201/2005”; European Union Directive 2010/63/UE). The Ethics Committee for Experimental Research of the Universidad Miguel Hernández approved the experimental protocols used.

### Mouse Transgenic Lines and Genotyping

For the study of the cortical cytoarchitecture and the *in vivo* electrophysiology experiments, we used wild-type and mutant littermates from the *Lis1/sLis1* line (described by Cahana et al., 2001; reviewed by Reiner et al., 2006). The mutant *Lis1/sLis1* mice (ICR background) are heterozygous for a deletion in the first exon of the mouse *Lis1* gene (*Pafah1b1* gene), which encodes for the LisH domain). This alteration prevents the normal dimerization of the protein and mimics a mutation previously described in a patient suffering from a mild form of lissencephaly (Fogli et al., 1999). Mutant *Lis1/sLis1* mice were identified by the amplification by PCR of a ≈750 bp DNA fragment amplified from ear tissue sample lysate. Primers used were the following ones: 3′GGT​GGC​AGT​GTT​GAG​ATG CCTAGCC5′ and 5′ GCA​TTC​CTG​TAA​TCC​AGT​ACC​TGG 3’. The PCR program consists of 35 cycles with a previous single initial denaturalization step of 94° for 5 min. Each cycle was composed of a denaturalization step of 94° for 40 s, a hybridization step of 60° for 45 s, and a polymerization step of 72° for 10 s. We used male animals in all experiments.

For the *in vitro* electrophysiology experiments, *Lis1/sLis1* mutant males were bred with GAD67-GFP females (C57BL6 background). GAD67-GFP line expresses GFP under the promoter of GAD67. GAD67 is a protein expressed in a wide range of cortical GABAergic interneurons (described in Tamamaki et al., 2003). Only GFP-expressing males were selected from the offspring of this breeding (as observed under a UV lamp after birth). Afterwards, with PCR genotyping (see above), we discriminated among those wild type and mutant for the LisH deletion in heterozygosis. We will refer to these animals as *Lis1/sLis1*-GAD67-GFP.

### Histological and Immunochemical Procedures

Thirty postnatal days (P30) wild type and mutant *Lis1/sLis1* mice (as indicated in Results) were anesthetized with urethane and transcardially perfused with 4% paraformaldehyde in PBS. Brains were removed and post-fixed in 4% paraformaldehyde/PBS overnight at 4°C, then placed in PBS and either cryoprotected in 30% sucrose or embedded in gelatin for frozen micro-sectioning. Brains were sectioned in coronal orientation at 40 μm on a freezing microtome (Microm). The sections were first treated with 0.3% H_2_O_2_ in PBS-T (PBS supplemented with 1% Triton X-100) for 30 min at room temperature to remove endogenous peroxidase activity for immunohistochemistry. Then, they were permeabilized and blocked using 1% Triton X-100, 1% BSA, and Lysine 0.1 M in PBS (Blocking solution) for 1 h at room temperature (RT) in a rocking table. Thereafter, the sections were incubated with rabbit polyclonal anti-calbindin (CB), calretinin (CR), and parvalbumin (PV) primary antibodies (SWANT Swiss Abs; 1:2000). The day after the incubation with the primary antibody, the sections were rinsed three times at room temperature and then incubated with the appropriate secondary biotinylated antibody for 1 h (goat anti-rabbit biotinylated 1:200 BA-1000/Vector). Next, the sections were washed with PBS-T at room temperature and incubated with Avidin-Biotin Complex for 1 h (1:300; ABC kit, Vector Laboratories CA-94010). The tissue was incubated with 1% 3,39-Diaminobenzidine (DAB; Vector Laboratories SK-4100) and 0.0018% H_2_O_2_ in PBS for the colorimetric detection.

To analyze cortical cytoarchitectonics, brains were fixed as described above were dissected and embedded in 4% to cut 40 µm sections in PBS with a vibratome (Leica VT1000). We used the following primary antibodies: anti-NeuN mouse monoclonal antibody (1:500; Chemicon/MAB377), anti-Cux1 rabbit polyclonal antibody (1:100; Santa Cruz/sc-13024), and anti-Foxp2 rabbit polyclonal antibody (1:500; Abcam/ab16046). The day after the incubation with the primary antibody, the tissue was incubated with the following secondary antibodies: goat anti-rabbit biotinylated 1:200 (BA-1000//Vector) and goat anti-mouse biotinylated 1:200 (BA-2020//Vector). For cresyl-violet staining, brain sections were treated following standard procedures.

### Cortical Cytoarchitectonics and Morphometric Analysis of the Primary Motor Cortex

The distributions of CB, CR and PV neurons in each area of the mouse primary motor cortex were determined using a rectangular lattice divided into five sampling tiers oriented tangentially to the pial surface. The width of each tier was adjusted so that the lattice sampled the whole extent of the cortical area present in the section. Sampling grids were then superimposed onto each tissue section to be analyzed. Using a 20X objective lens, the positions of the identified neurons present in each sampling tier that were positive for each antibody (CB, CR, or PV) were code-marked onto the bank of tiers used to sample each case. The absolute numbers of neurons in each horizontal tier of the grid were calculated and recorded. Cortical depth distribution histograms were later constructed with the number of neurons present in the tiers corresponding to Layer 2/3 (superficial layers) and the total number of cells for Layers 5 and 6 (deeper layers) relative to the total number of cells in superficial plus deep layers; this gives the percentage of each neuron population (CB, CR or PV) within superficial or deep layers. The same grid was used to sample each cortical area for each animal.

To analyze the overall cortical structure, bright-field images of the primary motor cortex stained with NeuN antibody were acquired at 2.5x magnification; the primary motor cortex was located according to the Allen Brain Atlas (http://www.brain-map.org/). The study of cortical layers was done with the software ImageJ (National Institutes of Health, USA). We measured the total thickness of the cortex and the thickness of individual layers in three to five sections per animal (three mice per genotype). Layers thickness was represented as the percentage calculated to the total cortical thickness (Sgado et al., 2013; Allegra et al., 2014). To quantify cell densities, we analyzed three sections per animal (wild type or *Lis1/sLis1*) using Image-J software (NIH, United States), and for each animal we used the mean values of these three sections to make statistical comparisons between genotypes.

### Recording and Analysis of Local Field Potentials in Anesthetized Mice

Local field potentials (LFP) were recorded in anesthetized mice using linear electrode arrays. Adult wild-type and mutant *Lis1/sLis1* mice (aged 3–6 months) were anesthetized with an initial dose of urethane (1.6 mg/g of weight) by intraperitoneal injection. Animals were placed in a stereotaxic device (Narisighe, Japan). Body temperature was maintained at 37°C using a heating pad. Also, Bupivacaine (0.5% in saline), an analgesic, was injected subcutaneously before surgery. The following coordinates were used for cranial trepanation and electrode array location, with respect to Bregma (using the Paxinos atlas as reference): −2 mm in anteroposterior axis, and ±1.5 mm in lateromedial axis, and 2.2/2.5 mm in the dorsoventral axis. We used linear electrode arrays (Michigan probe, NeuroNexus) containing 32 electrodes with a 50 µm separation. To confirm the proper location of the electrodes, the tip of the probes was impregnated with DiIl, and after the recording, the animals were perfused with PFA at 4%, the brains were removed, and 50 µm-thick slices were obtained. DiIl staining slices were selected and labeled with DAPI (10 μg/ml in PBS 1x solution).

Electrophysiological signals were acquired continuously at 20 kHz with Mc_Rack software, amplified, and digitized (ME64-PGA-MPA-System, Multichannel Systems, Reutlingen, Germany). Signals were passed through a notch filter (50 and 100 Hz) and high-pass filters (0.5 Hz) after down-sampling the acquisition frequency to 2.5 kHz. The power of the different frequency bands was obtained from the power spectrum of the signals calculated with a Fourier transform. The following frequency bands were used: delta, 0.5–4 Hz; alpha, 8–13 Hz; beta, 13–30 Hz; Gamma, 30–100 Hz. Band power was normalized to the low-frequency delta band. Signal processing, analysis, power spectra, and coherence calculations were performed with a MatLab code (Mathworks, MA, United States).

### *In vitro* Electrophysiological Recordings

Cortical slices (350 μm thick) were prepared from 28–32 days-old male wild type and mutant *Lis1/sLis1*-GAD67-GFP mice, using standard methods used in our laboratory (Rovira and Geijo-Barrientos, 2016; Sempere-Ferrandez et al., 2018). The slices were oriented coronally and included the primary motor cortex (at 1.30 mm anterior to Bregma according to the mouse brain atlas by Paxinos and Watson, 2001). Animals were sacrificed by cervical dislocation and coronal slices were cut with a Vibratome (Leica VT1000) in ice-cold low calcium/high magnesium cutting solution (composition: 124 mM NaCl, 2.5 mM KCl, 1.25 mM PO_4_H_2_Na, 2.5 mM MgCl_2_, 0.5 mM CaCl_2_, 26 mM NaCO_3_H and 10 mM glucose; pH 7.4 when saturated with 95% O_2_ and 5% CO_2_). The slices were transferred to artificial cerebrospinal fluid (ACSF; composition: 124 mM NaCl, 2.5 mM KCl, 1.25 mM NaPO_4_H_2_, 1 mM MgCl_2_, 2 mM CaCl_2_, 26 mM NaCO_3_H, and 10 mM glucose; pH 7.4 when saturated with 95% O_2_ and 5% CO_2_) where they were incubated at 37°C during 30 min and after that kept at room temperature until use.

For the recordings, individual slices were placed in a submersion-type chamber and perfused at a flow rate of 3–5 ml/min with ACSF at 32-34°C. Intracellular recordings were obtained with patch electrodes using the whole-cell configuration. Electrodes were made from borosilicate glass (1.5 mm o.d., 0.86 mm i.d., with inner filament) and had a resistance of approximately 3–5 MOhm when filled with the intracellular solution. An intracellular solution based on potassium gluconate (composition: 130 mM potassium gluconate, 5 mM KCl, 5 mM NaCl, 5 mM EGTA, 10 mM HEPES, 2 mM Mg-ATP, 0.2 mM Na-GTP; pH 7.2 adjusted with KOH; 285–295 mOsm) was used. All neurons were recorded under visual control using an upright microscope (Olympus BX50WI, Olympus American Inc.) equipped with epi-fluorescence, Nomarski optics, and a water immersion lens (40×). Only GFP-positive cells located in layers 2–6 were recorded. The proportion of FS and non-FS GABAergic interneurons was around 42% and 58% respectively in *Lis1/sLis1*-GAD67-GFP wild type and mutant animals, proportion quite similar to the one described in the GAD67-GFP line (Tamamaki et al., 2003). Our inclusion criteria for FS GABAergic interneuron group were the following: short action potentials (<0.4 ms measured at half action potential peak amplitude) with deep after-hyperpolarization (AHP), low input resistance (<280 MΩ), and high-frequency, non-adapting firing in response to depolarizing current pulses.

Voltage and current recordings were obtained with a two-channel Multiclamp 700B amplifier (Axon Instruments, Molecular Devices, United States), low-pass filtered at 4 kHz and digitized at 20 kHz with a 16-bit resolution analog to digital converter Digidata 1322A (Axon Instruments, Molecular Devices, United States). Once the recording was stable, the amplifier was switched to current-clamp mode to measure the spontaneous resting membrane potential. Then, if necessary, the membrane potential was adjusted to −70 mV with DC current injection, and the responses to a series of hyper- and depolarizing current pulses were recorded. Spontaneous excitatory postsynaptic currents (sEPSCs) were recorded by holding the membrane potential at −70 mV (Cl^−^ Eq potential ≈ −68 mV). The pipette liquid junction potential was estimated at −10 mV (using the junction potential calculator tool of the pClamp software); the series resistance (average value ∼20 MΩ; in all recordings was below 40MΩ) was monitored throughout the recording and compensated on-line only in current-clamp recordings using the “bridge balance” tool of the amplifier. Pulse generation and signal acquisition were controlled by the pClamp 9.2 software (Axon Instruments, United States).

### Immunohistochemistry of Human Brains

We have studied the dorsal part of the motor cortex (area M1, Brodmann’s area 4) in brains from a human lissencephalic patient (a female of 12 years old with a mutation in the chromosome 17p13.3, within the lissencephaly critical region). This brain was donated for research studies and medical teaching to the Anatomy Department of Miguel Hernandez University. After careful extraction, the brain was perfused through carotid and basilar arteries with 4% paraformaldehyde in PBS (3 L during 30 min). While the right motor cortex was completely lissencephalic, the left motor cortex was less affected with some normal gyri in its dorsal and medial cortex. We selected cortical blocks of dorsal motor areas from both hemispheres to be processed for sectioning at 40 μm on a freezing microtome (Microm). Nissl-staining was performed in alternative sections after mounting in slides. For immunohistochemistry, free-floating sections were first treated with 0.3% H_2_O_2_ in PBS-T (PBS supplemented with 1% Triton X-100) for 30 min at room temperature to remove endogenous peroxidase activity processed as described above. Immunohistochemistry was performed using polyclonal anti-calbindin (CB), calretinin (CR), and parvalbumin (PV) primary antibodies (SWANT Swiss Abs; 1:100). After immunoreaction with diaminobenzidine substrate (Sigma-Aldrich), the sections were mounted in slides and coverslip with Eukitt (Sigma-Aldrich).

### Statistical Analysis

Data are shown as mean ± s.e.m and sample size. Statistical comparisons were done with either Student’s t-test or Mann-Whitney rank-sum test (when the sample did not pass the normality test and/or the equal variance test) using SigmaStat 3.2 software (Systat Software Inc. United States). Differences were considered significant when the *p*-value was <0.05. In the figures, significant differences are shown with asterisks: *p < 0.05, **p < 0.01, and ***p < 0.001.

## Results

### Cytoarchitectonics of the Primary Motor Cortex and Distribution of PV, CB, and CR Positive Neurons in *Lis1/sLis1* Mice

**FIGURE 1:**
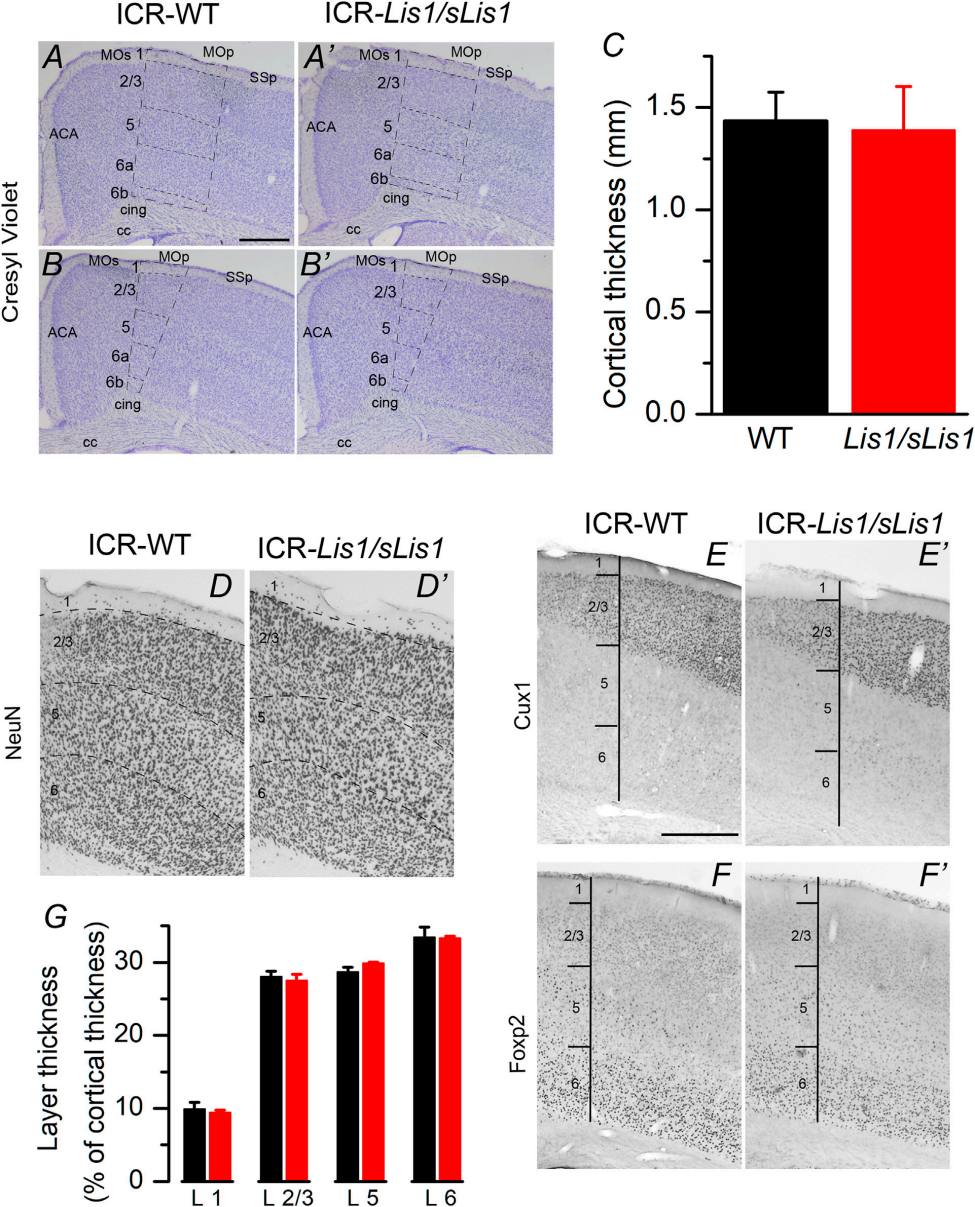
Cytoarchitectonics of the primary motor cortex of the wild type and mutant *Lis1/sLis1* mice. **(A–B)** Representative cresyl-violet staining of the primary motor cortex from wild type **(A,B)** and mutant (A′, B′) mice. Cortical layers indicated with dotted lines. ACA: anterior cingulate cortex, CC: corpus callosum, cing: cingulum MO: motor cortex, SS: somatosensory cortex. Section are from two antero-posterior levels (A, A′ anterior; B, B′ posterior). Scale bar in A: 0.5 mm. **(C)** Total thickness of the primary motor cortex measured from the cresyl-violet staining from wild type (black; n = 3 animals) and *Lis1/sLis1* (red; n = 3 animals) animals. **(D)** NeuN staining of the primary motor cortex of the wild type (D) and mutant (D′) mouse. **(E–F)** Staining of cortical layers using specific markers (Cux1, layer 2/3; FoxP2, layer 6). **(G)** relative thickness of the primary motor cortex layers from wild type (black; n = 3 animals) and *Lis1/sLis1* (red; n = 3 animals). To calculate the total thickness of the cortex and the thickness of cortical layers we measured these values in three stained slices in each animal (wild type or *Lis1/sLis1*) and used the mean values from these three slices to make comparisons between genotypes.

We first studied the impact of LisH deletion on the general structure of the primary motor cortex because the disruption of the neocortical structure is a classical hallmark associated with *LIS1* dysfunction. Experiments were performed at 30 postnatal days when the development of the cortical inhibitory neurons is complete. By doing a cresyl-violet (Nissl) staining and immunocytochemical staining against NeuN in the frontal (primary motor cortex), we detected a similar cortical thickness, neuron density, and layer structure in both genotypes (Figures 1A–D). We established the limits between layers from the Allen Brain Atlas’ data (www.brain-map.org), but to study with greater detail the layering of the primary motor cortex, we used specific markers for layers 2/3 (Cux1; Figure 1E) and layer 6 (Foxp2; Figure 1F). We did not identify a distinct layer 4 because the primary motor cortex of the mouse is agranular. However, recently, an extremely thin layer of pyramidal neurons with properties and connectivity similar to layer 4 neurons of somatosensory areas has been described (Yamawaki et al., 2014). The total thickness of the cortex and the relative thickness of layers 2/3, 5, and 6 were similar in wild-type and mutant cortices (Figures 1C,G; data compared with the Student’s t test). Neuronal density in cortical layers was also measured from NeuN stained sections, and no statistically significant differences were found between wild type and *Lis1/sLis1* mice: layer 2/3: wild type 3251 ± 59, Lis1/sLis1, 3194 ± 101; layer 5: wild type 2325 ± 28; Lis1/sLis1 2340 ± 106; layer 6: wild type 3217 ± 122; Lis1/sLis1 3588 ± 219 (values measured in 3 wild type animals and 3 Lis1/sLis1 animals and given as cells/mm2; Student’s t test, *p* > 0.4 in all cases). These results suggest that the overall structure of the primary motor cortex was identical in wild-type and *Lis1/sLis1* mice.

**FIGURE 2:**
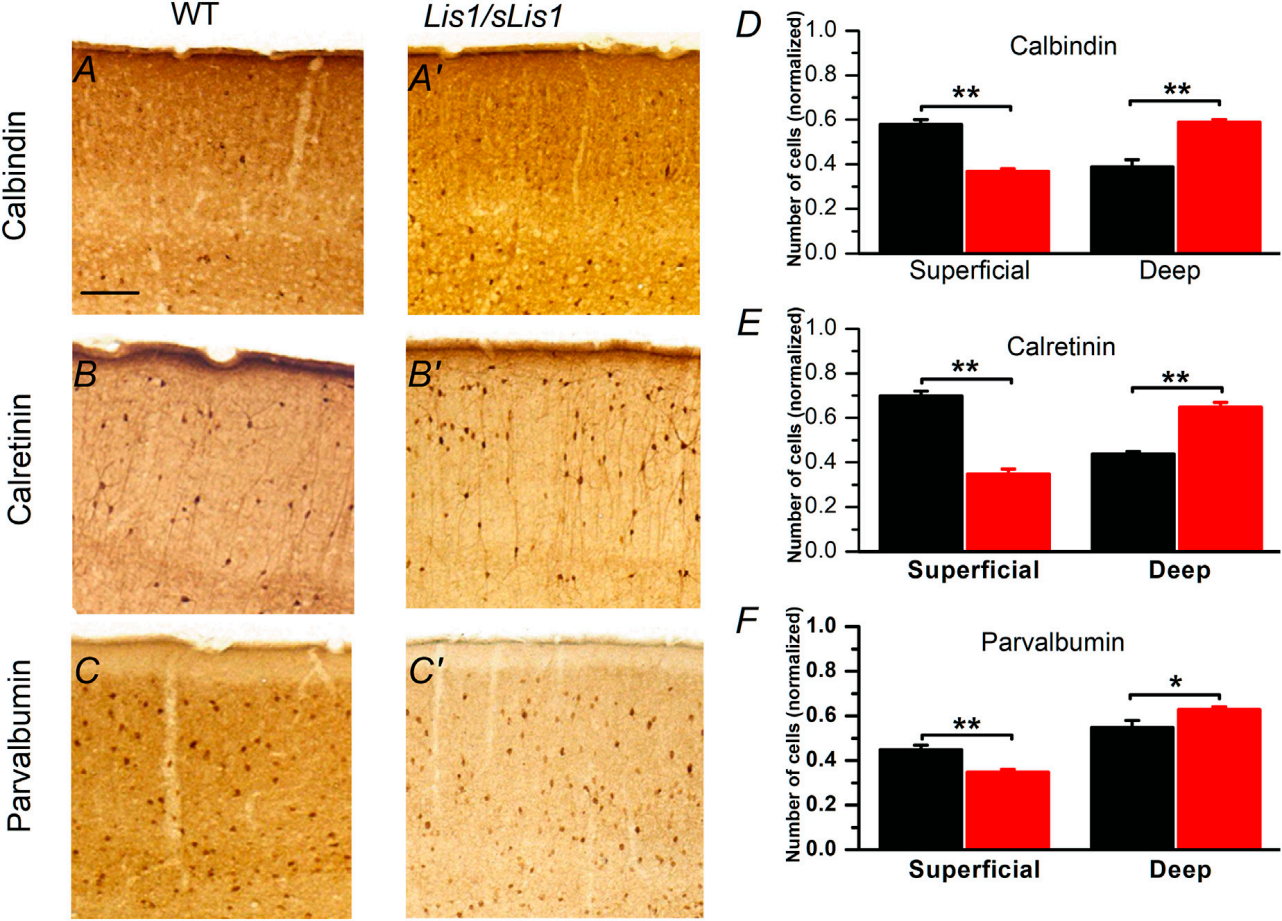
Distribution of calbindin, calretinin and parvalbumin positive cells in P30 mouse primary motor cortex of wild type and mutant *Lis1/sLis1* mice. **(A–C’)**. Immunohistochemical staining for calbindin (CB), calretinin (CR) and parvalbumin (PV) in wild type **(A–C)** and mutant **(A’–C’)** cortex. Scale bar in A (100 μm) applies to **(A–C’)**. **(D–F)** Normalized number (respect to the total cell number of each type) of CB, CR and PV positive cells in superficial and deep layers in wild type (black; n = 4) and mutant mice (red; n = 4). Superficial layers correspond to layer 2/3 while deep layers correspond to layers 5 and 6. The division of the cortical area between superficial and deep layers was based on the actual layering of the cortex and was done only as a way to count neurons. *p < 0.5, **p < 0.01, Student’s t-test.

The laminar pattern of the neocortex is determined by the top-down positioning of the different subtypes of excitatory pyramidal neurons, which are the more numerous neurons in the neocortex. GABAergic interneurons do not contribute decisively to the laminar pattern of the neocortex but play a crucial role in the cortical processing of information. However, given that Cahana et al. (2001) described the presence of neuronal migration deficits in the *Lis1/sLis1* mutant, it is possible that these deficits could alter the disposition of inhibitory neurons in the mature cortex. In the *Lis1/sLis1* mouse the GABAergic interneuron cell density in the primary motor cortex (measured from GAD67 positive cells in the *Lis1/sLis1*-GAD67-GFP mouse line, see methods) was similar in wild type and mutant mice (cells/mm^2^ ± sem, n = 4): controls, 253 ± 30; Lis1/sLis1, 183 ± 50). Since the overall density of GABAergic interneurons in the primary motor cortex was similar in wild type and mutant animals we tested the possibility that it was the distribution of GABAergic interneurons across cortical layers what could be altered in the *Lis1/sLis1* mouse, instead of the total number of GABAergic interneurons. To check this possibility, we have studied by immunolabeling the relative distribution among cortical layers of the somas of calbindin (CB), calretinin (CR), and parvalbumin (PV) positive GABAergic interneurons, which are well-defined, overlapping, groups of interneurons of the mammalian neocortex (Hendry and Jones 1991; Rogers 1992; Tremblay et al., 2016). The relative distribution of these interneurons in the superficial (layer 2/3) and deep (layers 5 and 6) layers was severely altered in the primary motor cortex of the mutant mice (Figure 2). A significant increase in the relative number of CB, CR, or PV-positive neurons was detected in the deep layers of mutant primary motor cortex; this increase was coincident with a significant decrease of the relative number of neurons of these groups in the superficial layers (Figure 2). Interestingly, this altered distribution of cortical CB and CR positive cells in the mutant cortex reversed the normal gradient of these cells between superficial and deep layers found in wild type animals (Figures 2D,E); in contrast, the distribution of PV cells in the mutant cortex conserved the gradient between superficial and deep layers observed in WT cortex, but this gradient was enhanced (Figure 2F). Overall, the deletion of the LisH domain disrupted the distribution of CB, CR, and PV expressing GABAergic interneurons in the mutant primary motor cortex.

### Cortical Electrical Activity in the Primary motor Cortex of Anesthetized *Lis1/sLis1* Mice

Oscillations characterizing cortical electrical activity are rhythmic patterns of activity and are considered the functional substrate underlying cognitive and behavioral processes. These electrical oscillations result from the synchronized activity of the different neuronal components in the cortical circuits, and GABAergic interneurons are critical players of this synchronous activity. Therefore, we hypothesized that the altered positioning of different interneuron subtypes across cortical layers in mutant mice might have disrupted the oscillatory electrical activity in the primary motor cortex.

**FIGURE 3:**
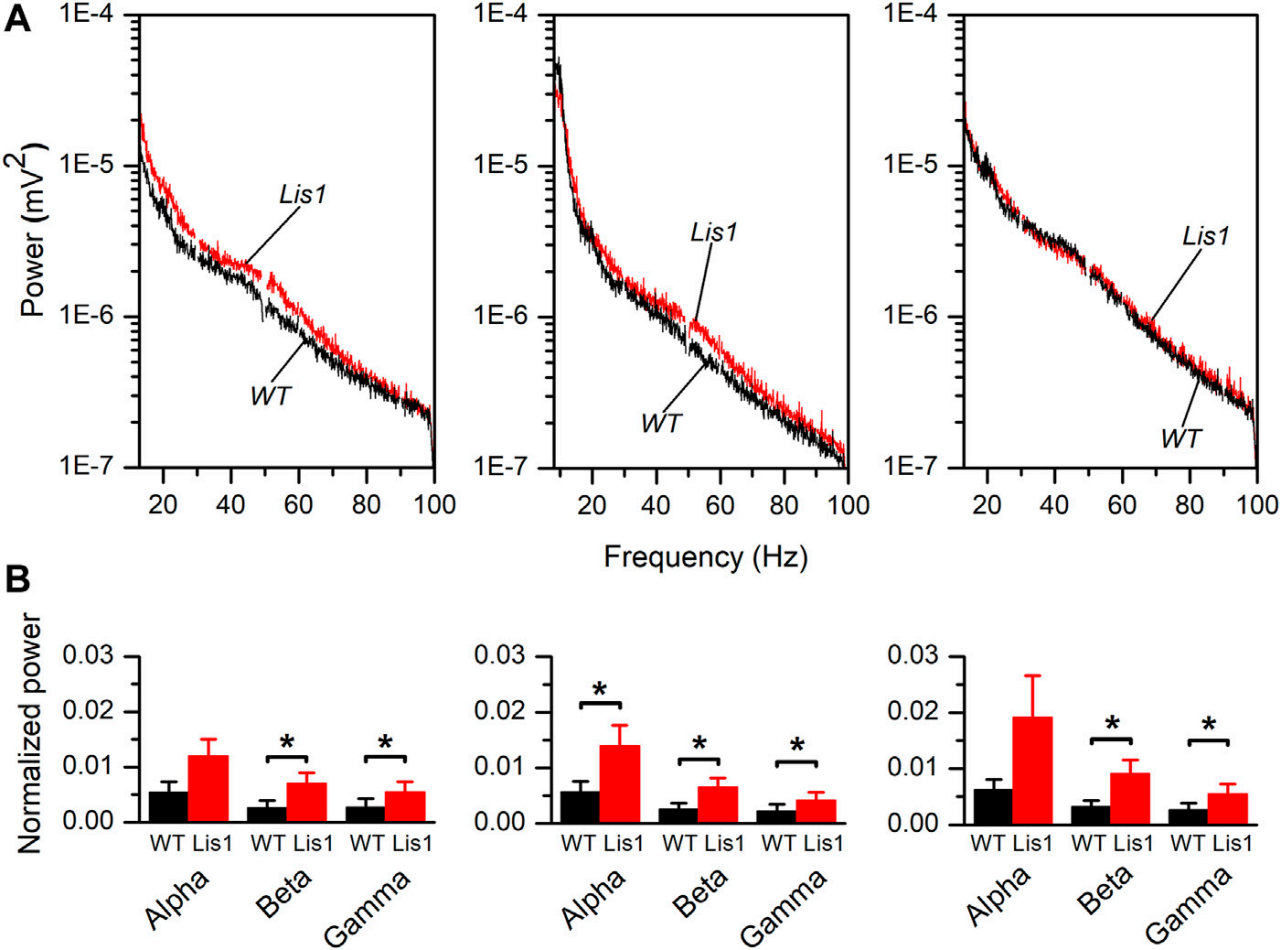
Frequency bands in the electrical activity recorded *in vivo* in the primary motor cortex of wild type and mutant *Lis1/sLis1* mice. (**A**). Averaged power spectra of the LFP recorded in the primary motor cortex from layer 2/3 (left panel), layer 5 (center panel), and layer 6 (right panel) in wild type (black; n = 8) and mutant (red; n = 11) animals. The area between 49.5 and 50.5 Hz has been removed to avoid the artifact that appears due to the 50 Hz notch filter. The frequency bands were: alpha: 8–13 Hz, beta: 13–30 Hz, gamma: 30–100 Hz. (**B**). Power values of each frequency band normalized for the power of the delta band in layer 2/3 (left panel), layer 5 (center panel), and layer 6 (right panel). Normalization of the values obtained from each animal was performed before calculating the group means. To calculate the average normalized power value, the whole frequency range of each band was used. Averaged values are given as mean ± s.e.m. *p < 0.5, Student’s t-test.

To confirm this hypothesis, we performed *in vivo* electrophysiological recordings of local field potentials (LFP) by placing a multi-electrode probe in the primary motor cortex of adult (30 days postnatal) urethane-anesthetized wild-type, and mutant *Lis1/sLis1* mice. We first analyzed the power of the frequency bands of the cortical activity recorded in layers 2/3, 5, and 6, covering the alpha (8–13 Hz), beta (13–20 Hz) and gamma (30–100 Hz) bands (Figure 3A). Overall, the power of the oscillatory electrical activity was higher in mutant mice. To compare the power of the activity recorded in different animals, the power values were normalized with respect to the power of the delta band (0–4 Hz) to confirm that the differences in activity were not due to differences in the level of anesthesia of each experimental animal. The power of the delta band was similar in wild type and mutant mice (layer 2/3: wild type 0.01 ± 0.004 mV^2^, mutant 0.005 ± 0.002 mV^2^; layer 5: wild type 0.077 ± 0.015 mV^2^, mutant 0.066 ± 0.015 mV^2^; layer 6: wild type 0.076 ± 0.013 mV^2^, mutant 0.075 ± 0.019 mV^2^. In all cases the *p*-value were >0.05). This normalized power of activity recorded in layers 2/3, 5, and 6 in wild-type and mutant mice can be observed in Figure 3B. The power of the beta and gamma bands were found significantly larger in mutant animals in all the layers analyzed; in contrast, the power of the alpha band was significantly larger in mutant animals only in layer 5 (Figure 3B). Therefore, cortical circuits from layers 2/3, 5, and 6 produce aberrant synchronic patterns of electrical oscillatory activity. This is relevant since electrical oscillatory activity at high frequencies, especially in the gamma band, is strongly shaped by fast-spiking GABAergic interneurons activity (Kawaguchi et al., 2019).

**FIGURE 4:**
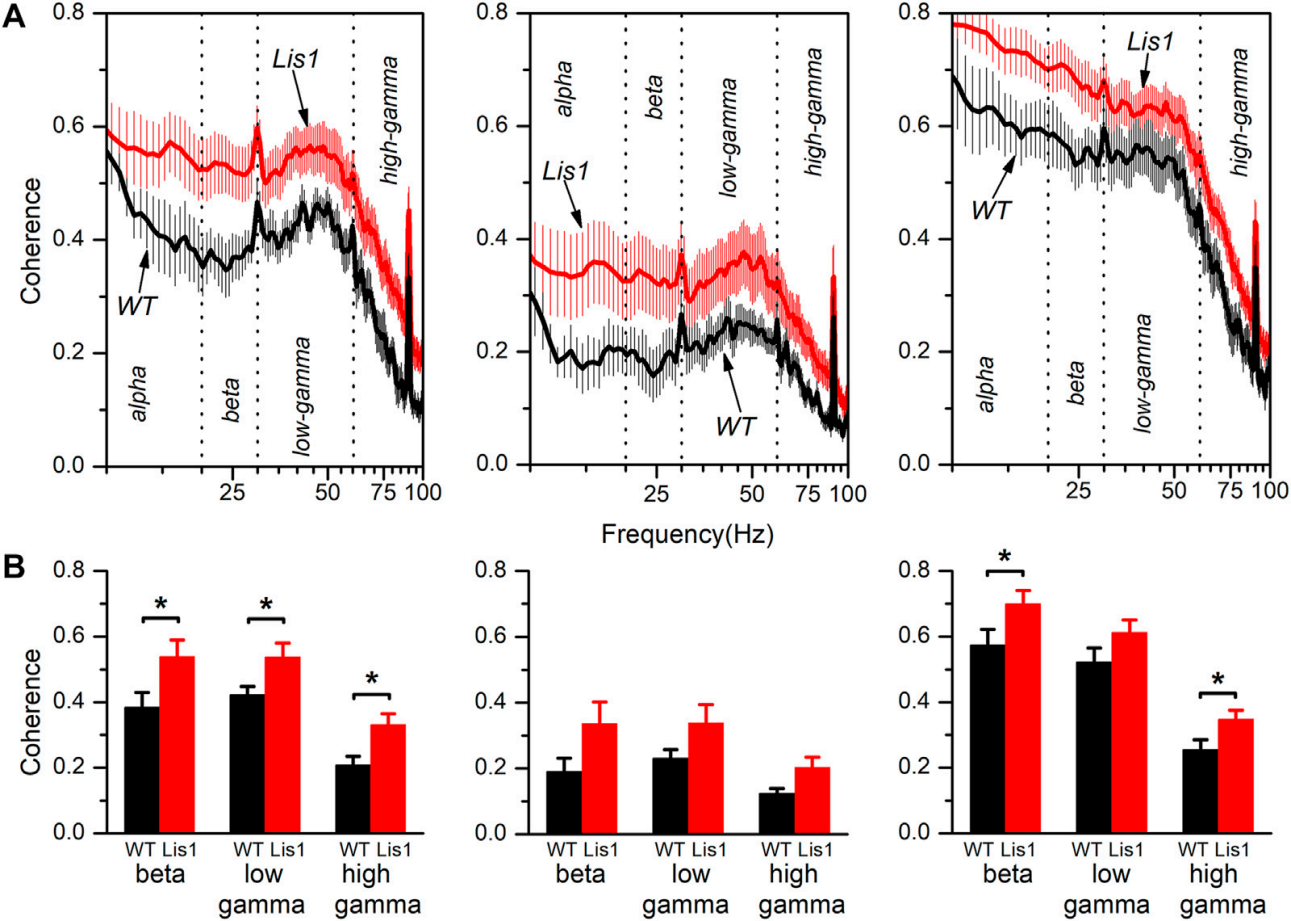
Spectral coherence between layers of the electrical activity in the primary motor cortex of wild type and mutant *Lis1/sLis1* mice. **(A)** The figure shows the averaged coherence along the frequency spectrum between layer 2/3 and 5 (left panels), layer 2/3 and 6 (middle panels), and layer 5 and 6 (right panels) of the electrical activity recorded in wild type (black traces; n = 8) and mutant mice (red traces; n = 11). **(B)** Comparison of the coherence among genotypes in different frequency bands between layers 2/3 and 5 (left panels), layers 2/3 and 6 (middle panels), and layers 5 and 6 (right panels). The frequency spectrum was divided into the following bands; alpha (8–13 Hz), beta (13–20 Hz), low-gamma (30–60 Hz), and high gamma (60–100 Hz). All data are presented as a mean ± s.e.m. *p < 0.5, Student’s t-test.

The neocortex is functionally organized in cortical columns. In the primary motor cortex, each cortical column is composed of the interconnected local circuits from layer 2/3, layer 5, and layer 6. Again, the alteration of GABAergic interneurons positioning across cortical layers and the inability of that layers to produce normal patterns of synchronic oscillatory activity led to the hypothesis that functional connectivity along the cortical columns must also be impaired. To corroborate that, we took advantage of the simultaneous recordings obtained from each layer to study the coherence of the oscillatory electrical activity between the different layers in the primary motor cortex. Figure 4A shows the spectral coherence in the 8–100 Hz range between layers 2/3 and 5 (left panels), layers 2/3 and 6 (middle panels), and layers 5 and 6 (right panels) for wild type and mutant mice. Overall, the coherence values among layers were higher and relatively constant at low and intermediate frequencies (8–60 Hz) and sharply decreased approximately from 60 to 100 Hz. Also, coherence values among layers 2/3-5 and layer 5–6 are higher respect values among layers 2/3-6, which is consistent with a previous study describing the interlayer glutamatergic connectivity in the primary motor cortex (Weiler et al., 2008).

Interestingly, the coherence values between layers were on average higher in the mutant animals. The coherence values for each frequency band (alpha: 8–13 Hz, beta: 13–20 Hz, low-gamma: 30–60 Hz, and high gamma: 60–100 Hz) are compared in Figure 4B. We decided to split the gamma frequency spectrum in the previously mentioned ranges because of the apparent different behavior of coherence within these gamma intervals. By comparing the average coherence values between layers 2/3 and 5, we observed a significant increase in all three frequency bands (beta, low, and high gamma) in mutant animals. Also, the coherence between layers 5 and 6 was significantly higher in the beta and high gamma bands in mutant animals. In contrast, we detected no significant differences in coherence value among layers 2/3 and 6 nor layers 5 and 6 in the low gamma band.

The inability of local circuits at the layer level to generate normal patterns of oscillatory electrical activity indicates a lack of synchronicity among the different neuronal components. Also, the alteration in the coherence among different cortical layers shows aberrant synchronicity among them. Overall, the data from LFP *in vivo* recordings suggest a general impairment of the functional connectivity in the primary motor cortex of the mutant mice.

### Electrophysiological Properties of Fast Spiking GABAergic Neurons in the *Lis1/sLis1*-GAD67-GFP Cortex

*In vivo* recordings showed a general alteration in the functional connectivity in the mutant mice’s primary motor cortex, mainly affecting the oscillatory electrical activity patterns in the medium-high frequency spectra, which is primarily attributed to Fast-spiking (FS) GABAergic interneurons activity. Thus, we hypothesized that the altered integration of FS GABAergic interneurons might partially explain this functional connectivity alteration to the cortical circuits. To answer our hypothesis, we decided to study the excitatory synaptic input to FS GABAergic interneurons by performing whole-cell *in vitro* electrophysiological recordings in the primary motor cortex in brain slices at 30 postnatal days, when cortical circuits are already established.

**FIGURE 5:**
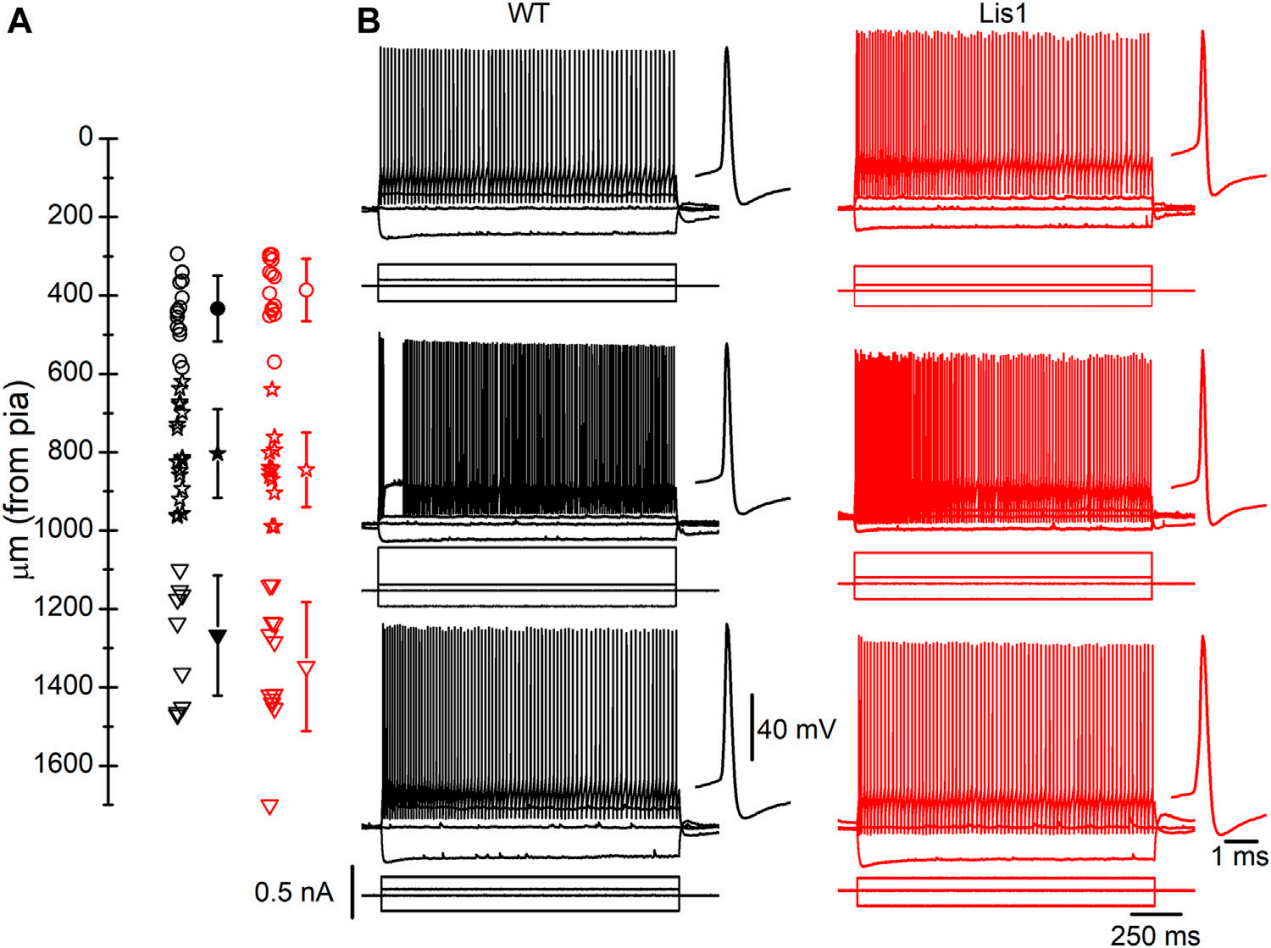
Fast-spiking (FS) interneurons recorded in *Lis1/sLis1*-GAD67-GFP wild type and mutant mice primary motor cortex. **(A)** Position of the soma of all recorded FS interneurons within the cortical layers; Open circles, stars, and triangles represent layers 2/3, 5, and 6 individual positions of FS interneurons for wild type (black) and mutant (red) mice. The filled symbols indicate the average position of the soma of the recorded FS interneurons in each cortical layer of wild type (layer 2/3 433.6 ± 21.58 µm, n = 15; layer 5 803.06 ± 26.63 µm, n = 18; layer 61,267.9 ± 48.45 µm, n = 10) and mutant mice (layer 2/3 386.6 ± 21.27 µm, n = 14; layer 5 845 ± 27.54 µm, n = 12; layer 61,346.9 ± 46.32 µm, n = 12). The limits between layers were placed as follows: layer 1, pia surface to 300 μm; layer 2/3, 300–550 μm; layer 5, 550–950 μm; layer 6, deeper than 950 μm; these limits were based on the cortical layers depicted in the Allen Brain Atlas (www.brain-maps.org) and our data in Figure 1. **(B)** Examples of the responses to hyper- and depolarizing current pulses of FS interneurons. Top to bottom: a representative recording of layer 2-3, layer 5, and layer 6 FS interneurons. The first action potential of the train is shown, at a larger time scale, next to each response to the supra-threshold current pulses. Scale bars apply to all panels. The resting membrane potential of the FS interneurons shown were (left to right, top to bottom): −71.5 mV, −63 mV, −58 mV, −70 mV, −64 mV, −74 mV.

GABAergic interneurons are about 20% of total neurons in the neocortex, and approximately half of them are FS interneurons (Tremblay et al., 2016). To facilitate the identification of FS interneurons in brain slices, we bred the *Lis1/sLis1* line with the GAD67-GFP line, which expresses the green fluorescent protein (GFP) under the promoter of GAD67, a specific marker for GABAergic interneurons (more details in the methods section). We selected those GFP-expressing animals that were also genotyped to determine those wild type and mutant for the LisH deletion from the offspring. GFP-expressing GABAergic interneurons were recorded in all the motor cortex layers in wild-type and mutant *Lis1/sLis1*-GAD67-GFP animals. FS GABAergic interneurons were discriminated based on their easily identifiable electrical properties (Kawaguchi and Kubota, 1997; Ascoli et al., 2008; Kepecs and Fishell, 2014; Tremblay et al., 2016. FS inclusion criteria described in the methods section). The position of the soma of the recorded FS GABAergic interneurons in the cortex of wild-type and mutant mice is shown in Figure 5A. The electrophysiological properties of the FS neurons recorded in layers 2/3, 5, and 6 in wild-type and mutant mice are provided in Table 1. Overall, no relevant differences were found in passive electrophysiological properties except the peak amplitude of the action potential, which was significantly smaller in layer 2/3 of mutant FS neurons. In addition, the threshold for spike firing was more negative in layer 6 mutant FS neurons.

**TABLE 1:** Electrophysiological properties of Fast-Spiking interneurons in *Lis1/sLis1-GAD67-GFP* wild type and mutant mice Values are shown as mean ± s.e.m. and the number of FS interneurons; the range is given in parenthesis. The resting membrane potential (E_m_) was measured shortly after breaking into the whole-cell mode and obtaining a stable recording. The input resistance (IR) was measured from the responses to small hyperpolarizing current pulses applied from E_m_. The membrane time constant (τ_m_) was measured from the fitting of an exponential to the charging phase of the voltage response to small hyperpolarizing current pulses. Action potential peak amplitude (AP peak) was measured from the threshold, and the action potential duration (AP dur) was measured at 50% of the peak amplitude. Rheobase is the minimum amplitude of a depolarizing current pulse of 750 ms of the period necessary to reach the threshold for spike firing. Statistical comparisons made with the Mann-Whitney rank sum test.

	Layer 2/3		Layer 5		Layer 6	
	WT	*Lis1/sLis1*	WT	*Lis1/sLis1*	WT	*Lis1/sLis1*
E_m_ (mV)	−70.6 ± 0.97 n = 15 (−79.5–−66.0)	−68.1 ± 2.04 n = 13 (−76.0–−52.0)	−69.2 ± 1.64 n = 19 (−83.0–−57.0)	−70.7 ± 1.32 n = 13 (−76.0–−58.5)	66.6 ± 1.47 n = 13 (−77.0–−58.0)	−71.5 ± 1.71 n = 11 (−80.0–−57.0)
IR (MΩ)	83.0 ± 7.78 n = 13 (42.0–142.6)	82.8 ± 19.97 n = 11 (41.6–276.3)	111.8 ± 14.08 n = 15 (47.6–231.4)	91.0 ± 9.43 n = 13 (41.4–156.4)	99.9 ± 12.83 n = 14 (40.2–195.7)	101.1 ± 15.0 n = 9 (58.0–185.0)
τ_m_ (ms)	4.33 ± 0.51 n = 13 (2.37–9.39)	3.86 ± 0.43 n = 11 (2.03–7.39)	5.43 ± 0.96 n = 8 (3.2–11.7)	4.29 ± 0.47 n = 12 (1.9–6.4)	3.89 ± 0.48 n = 8 (1.15–5.18)	6.13 ± 1.36 n = 8 (2.58–14.41)
Threshold (mV)	−38.9 ± 1.09 n = 13 (−44.5–−31.2)	−41.4 ± 2.05 n = 13 (−53.6–−29.3)	−39.2 ± 2.03 n = 10 (−46.8–−25.5)	−42.9 ± 1.85 n = 13 (−57.3–−33.1)	−33.9 ± 1.75 n = 11 (−41.5–−26.5)	−48.3 ± 2.54***n = 11 (−62.0–−37.6)
AP peak (mV)	74.0 ± 2.15 n = 13 (59.2–89.2)	63.5 ± 7.25***n = 13 (49.1–73.2)	78.4 ± 2.29 n = 10 (68.1–88.0)	76.4 ± 2.91 n = 13 (57.26–90.0)	75.6 ± 3.50 n = 10 (58.6–91.5)	80.0 ± 3.85 n = 11 (48.8–94.3)
AP dur (ms)	0.18 ± 0.007 n = 13 (0.16–0.25)	0.19 ± 0.010 n = 13 (0.15–0.25)	0.19 ± 0.016 n = 10 (0.15–0.32)	0.17 ± 0.010 n = 13 (0.13–0.26)	0.19 ± 0.016 n = 11 (0.14–0.30)	0.23 ± 0.020 n = 11 (0.16–0.38)
Rheobase (pA)	270.0 ± 28.6 n = 12 (120–450)	230.8 ± 24.4 n = 13 (60–390)	200.0 ± 21.9 n = 18 (60–330)	193.8 ± 25.1 n = 13 (90–330)	205.4 ± 30.9 n = 13 (60–420)	163.6 ± 30.6 n = 11 (30–360)

**FIGURE 6:**
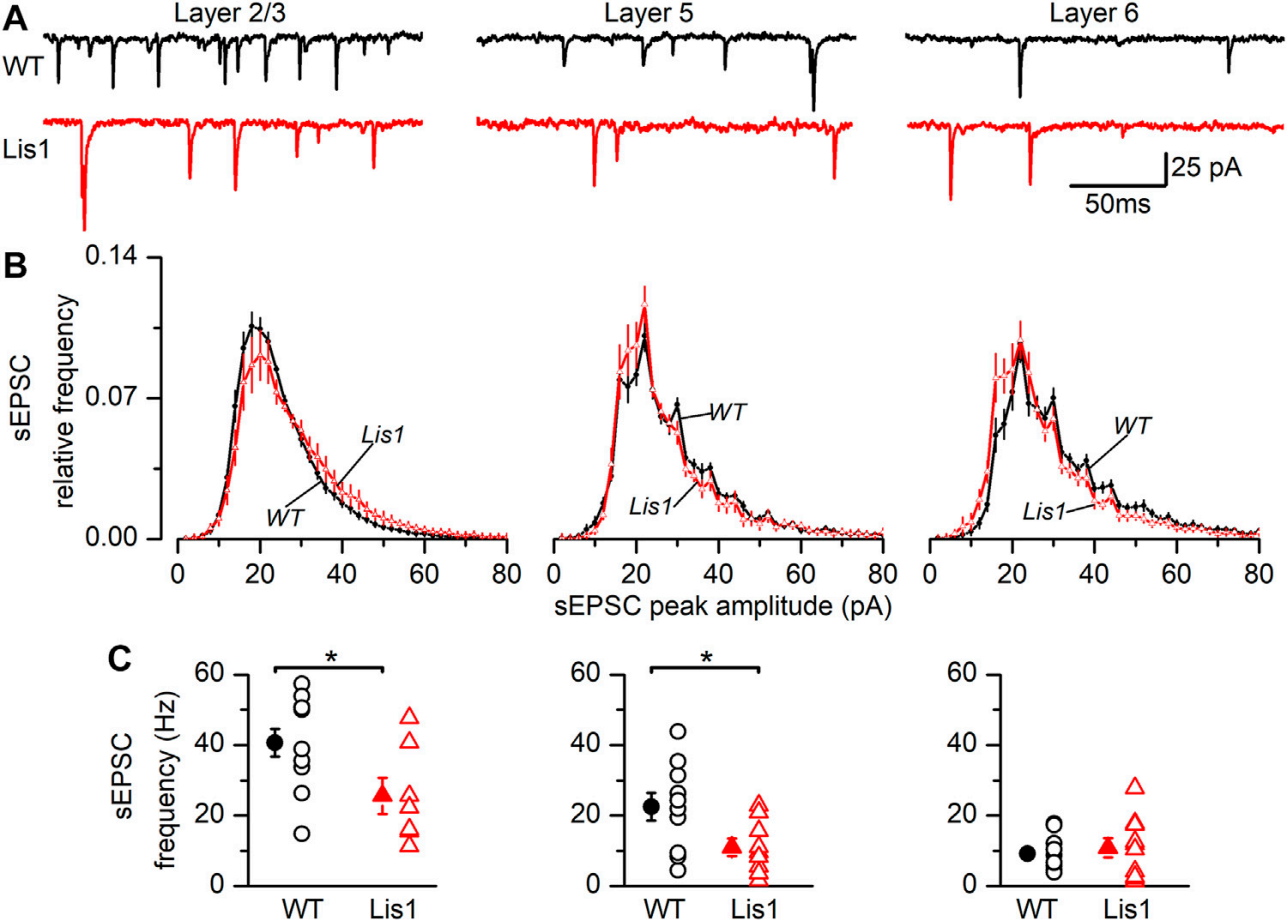
Properties of the sEPSCs recorded in Fast-Spiking (FS) interneurons in the GAD67-GFP-*Lis1/sLis1* wild type and mutant mice. The figure shows the properties of the sEPSCs of wild type (black) and mutant (red) FS interneurons recorded from left to right in layer 2/3, layer 5, and layer 6, respectively. **(A)** Sample traces of the membrane current where sEPSCs were detected. **(B)** Averaged relative frequency distribution of the peak amplitude of the sEPSCs (2 pA bins). **(C)** sEPSCs frequency values from FS interneurons. Panels **(B–C)** averaged values are given as mean ± s.e.m. (wild type n = 12, mutant n = 7; layer 5: wild type n = 10, mutant n = 9; layer 6: wild type n = 12, mutant n = 10). *p < 0.5, Student’s t-test.

To study the synaptic excitatory input in FS GABAergic interneurons, we characterized the spontaneous excitatory synaptic currents (sEPSC’s). (Figure 6). In layer 2/3 FS GABAergic interneurons the frequency of sEPSC was significantly lower in mutant animals, whereas the distribution of the sEPSC peak amplitude was shifted to larger values (Figures 6B,C, left panels). As a consequence of this shifted distribution, the mean peak amplitude of the sEPSC was larger in mutant neurons than in their wild type counterparts (wild type 22.72 ± 0.64 pA, n = 11; mutant 25.89 ± 1.18 pA, n = 7; *p* < 0.05, Student’s t test). In layer 5, sEPSC frequency was also lower in mutant neurons; however, no differences were detected in the distribution of the peak amplitudes (Figures 6B,C, middle panels) and the mean value of the peak amplitude was similar across genotypes (wild type 27.65 ± 0.73 pA, n = 10; mutant 29.32 ± 2.08 pA, n = 9; n.s.). Finally, no differences were observed in layer 6 (Figures 6B,C, right panels), including the mean value of the sEPSC peak amplitude (wild type 32.35 ± 2.02 pA, n = 12; mutant 32.21 ± 4.68 pA, n = 10). The time course of the sEPSC, measured by the time constant of the decay phase, was similar in wild type and mutant neurons from all layers: layer 2/3 wild type 1.21 ± 0.09 ms and mutant 1.33 ± 0.16 ms, layer 5 wild type 1.27 ± 0.06 ms and mutant 1.31 ± 0.15 ms and layer 6 wild type 1.85 ± 0.09 ms and mutant 1.72 ± 0.18 ms. In all cases, the differences were not statistically significant. Altogether, these results clearly point to an alteration of the excitatory input to FS GABAergic interneurons, and therefore an altered integration of FS GABAergic interneurons in the local cortical circuit.

**FIGURE 7:**
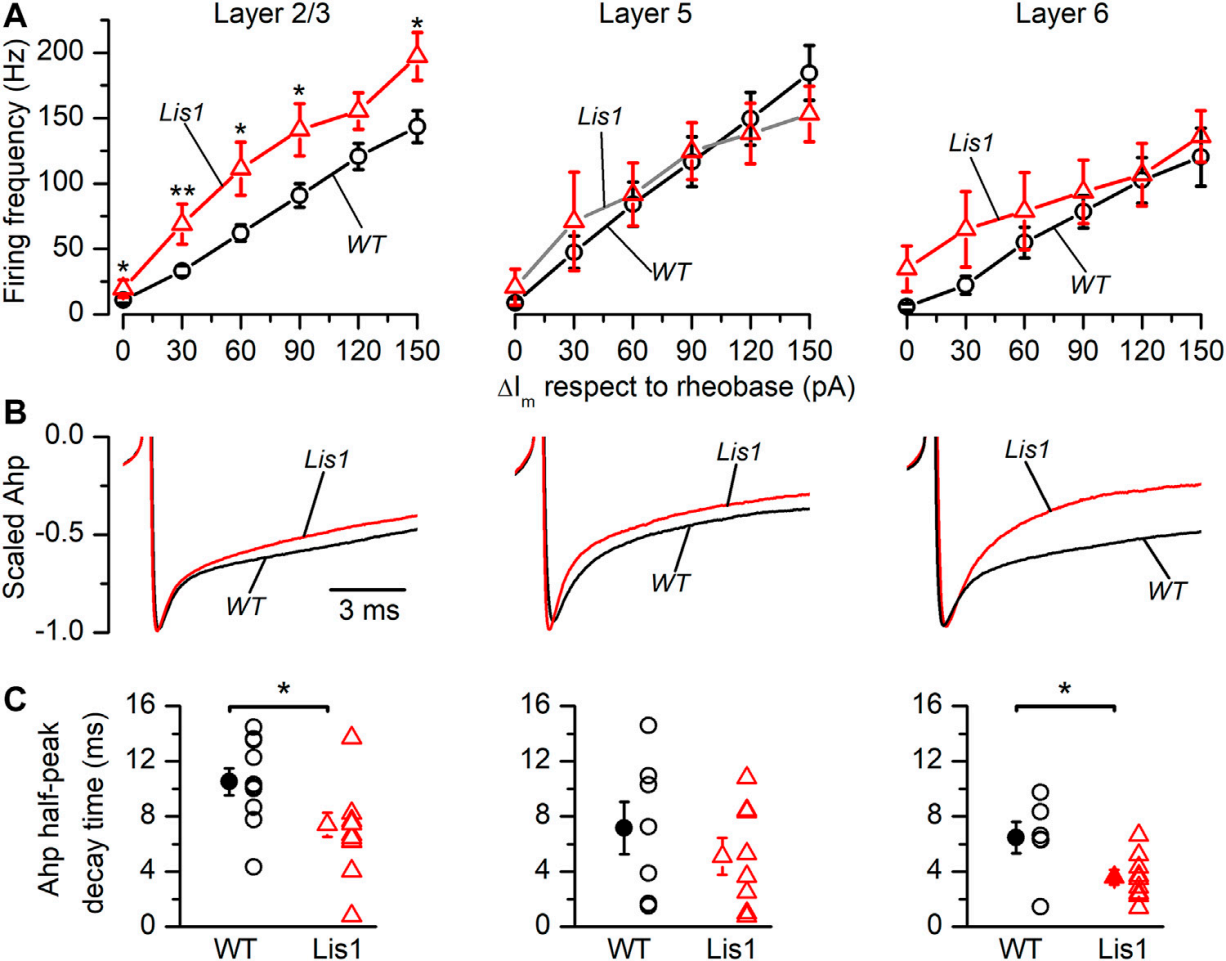
Firing frequency in response to depolarizing current pulses and properties of the action potential after-hyperpolarization (AHP) in Fast-Spiking (FS) interneurons in Lis1/sLis1-GAD67-GFP wild type and mutant mice. Panels **(A–C)** from left to right show layer 2/3, layer 5, and layer 6 FS interneurons values from wild type (black) and mutant (red) animals. **(A)** Relationship between the firing frequency and the strength of the depolarizing current pulses obtained in wild type (Layer 2/3 *n* = 10; Layer 5, *n* = 18; Layer 6, *n* = 14) and mutant FS interneurons (Layer 2/3, *n* = 12; Layer 5, *n* = 13; Layer 6, *n* = 12) using the same protocol as that shown in Figure 5B. The strength of the depolarizing current pulses is shown as current increments with respect to the rheobase. **(B)** Averaged time course of the AHP (wild type: Layer 2/3, *n* = 9; Layer 5, *n* = 8; Layer 6, *n* = 10. Mutants: Layer 2/3, *n* = 9; Layer 5, *n* = 9; Layer 6, *n* = 9). The traces are scaled between the threshold and the peak to compare the time course of the AHP. **(C)** Half-amplitude decay time of the action potential AHP of wild type and mutant FS interneurons.

It also has been proposed that oscillation frequency of oscillatory electrical activity, especially at medium-high frequency bands like the gamma band, are determined by the firing rate of FS GABAergic interneurons (Beierlein et al., 2003). Thus, we studied firing frequency in FS GABAergic interneurons in the different cortical layers. In Figure 7A are depicted the relationship between the firing frequency and the strength of the depolarizing current pulses. The size of the depolarizing current pulses is given as increments from the rheobase, which was similar in all groups of FS neurons (Table 1). FS neurons from layer 2/3 in mutant animals fired at higher frequencies in response to depolarizing current pulses Figure 7A. FS neurons from layer 6 in mutant animals also fired at significantly higher frequencies, but this difference was restricted to frequencies of 90 Hz or lower (Figure 7A). In contrast, no significant difference was observed in the firing frequency of FS neurons from layers 5 in response to depolarizing current pulses. The firing frequency during a square suprathreshold depolarizing current pulse is determined by several factors; one of these factors is the action potential after-hyperpolarization (AHP) and therefore differences in the AHP could cause changes in the action potential firing frequency. The repolarization phase of the AHP was faster in mutant neurons from layer 2/3 and 6 (Figures 7B,C) and the peak amplitude of the AHP (measured from the spike threshold) was smaller in layer 6 mutant neurons (Figure 7D). These differences in the amplitude and time course of the AHP could cause, at least in part, the differences in the frequency of the firing in response to depolarizing current pulses.

### Distribution of PV, CB, and CR Positive Neurons in Human Lissencephalic Cortex

**FIGURE 8:**
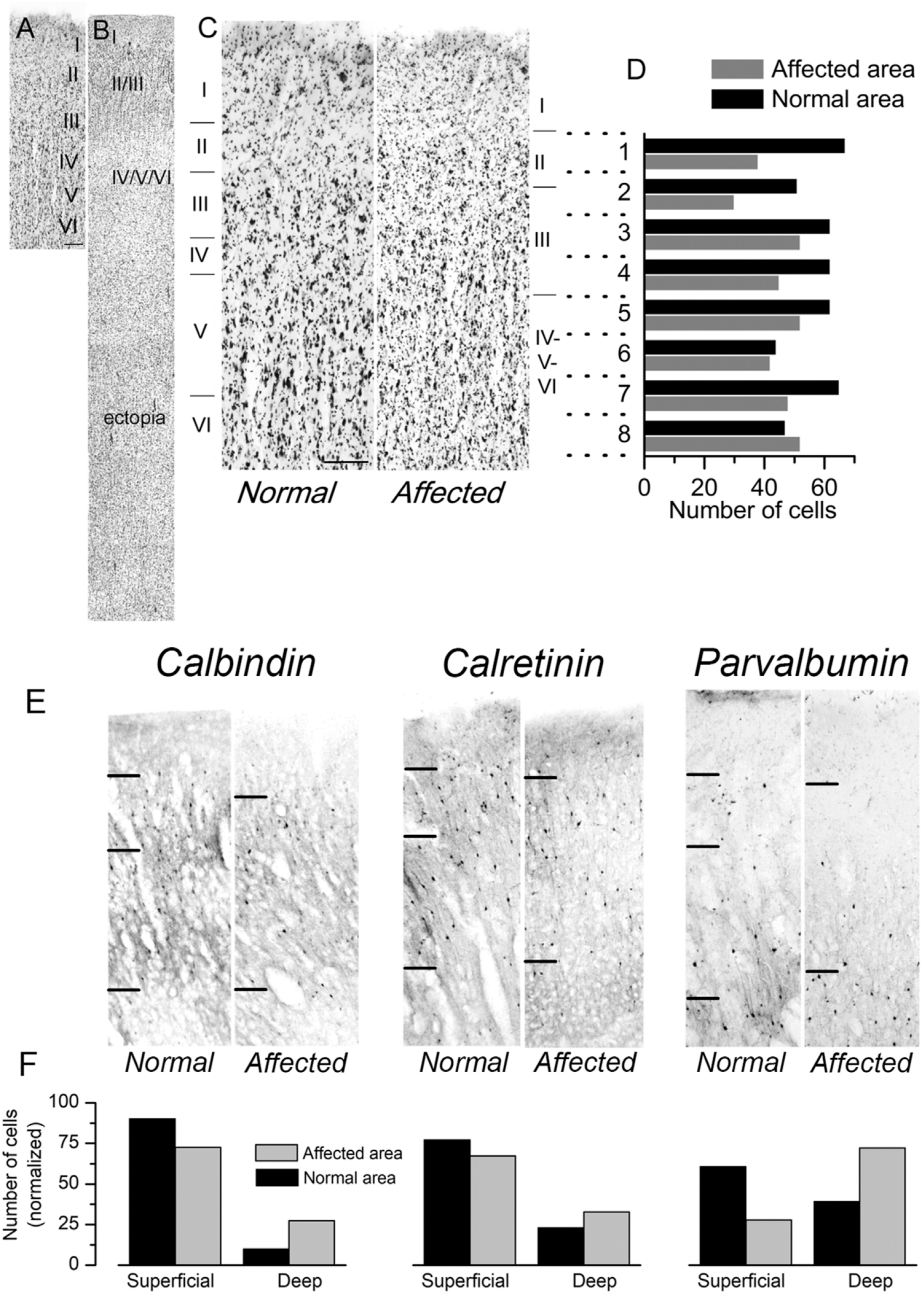
Cytoarchitectonics of the primary motor cortex in a case of human lissencephaly. **(A,B)** Nissl-stained sections of the dysplastic frontal cortex (primary motor cortex; B) and a normal area of the same cortical region **(A)**. The cortical layers are indicated on each panel. In the affected area **(B)**, a sizeable cortical ectopia is evident below the cortex. The normal area shown in panel A had approximately 4 mm in thickness. **(C)** Examples of the normal cortex and the affected cortex from the same subject are shown at a larger scale. Scale bar: 0.4 mm. In panels **(A–C)**, the cortical layers are indicated. **(D)** Cortical areas shown in panel C were divided into eight horizontal bands of the same thickness (dotted lines). The cells in each band were detected and counted with the Image-J software (NIH, USA). The number of cells detected in each horizontal band is shown in the plot. **(E)** Distribution of calbindin, calretinin, and parvalbumin-positive interneurons in the same patient’s regular and dysplasic cortical areas. Upper layer cortical interneurons (layers II and III) of the lissencephalic motor cortex showed an apparent reduction of interneurons (calbindin, calretinin, and parvalbumin-positive cells) compared to deep layers. **(F)** Count of calbindin, calretinin, and parvalbumin-positive interneurons (left, center, and right panels, respectively). The cell counting was made in superficial layers (I-IV), deep layers (V-VI), and the values are given are relative to the total number of positive cells in all layers. The values shown in each column are the average of three consecutive sections.

We had the opportunity to study a case of human lissencephaly and compare the results with the cortical structure of mutant *Lis1/sLis1* animals. The lissencephalic human brain studied (from a 12 years old female with a deletion in the chromosome 17p13.3, within the lissencephaly critical region) showed a partial lissencephaly (bilateral fronto-parietal lissencephaly and temporal pachygyria). The deletion present in this patient affected the *LIS1* gene, but we cannot discard that other close genes, such as *YWHAE* were also affected, although there have been described cases of microdeletions of *YWHAE* distal to the LIS1 gene (Schiff et al., 2010). This mutation in the human brain affect the whole gene, therefore only haploinsufficiency should be considered. Mutations restricted to the LisH domain may generate more severe phenotype since dominant negative effect factors may be associated. The left hemisphere was less affected: it had not macroscopic nor microscopic heterotopic band underneath the cortical grey matter and histologically all layers were recognizable, In comparison to the less affected left primary motor cortex, the right primary motor cortex (Brodmann’s areas 8, 6 and 4 did not show gyri and frontal opercular region was not formed) showed a reduction of cortical thickness and disorganization of the cortical layers (Figure 8), which made their identification difficult, in the presence of a large subcortical band of neuronal ectopia (Figures 8A,B). The left motor cortex was less affected with some identifiable gyri in its dorsal and medial cortical areas (superior and interhemispheric regions of Brodmann’s 6 and 4 areas). Although the layer distribution of neurons and cortical thickness were normal in the left dorsal primary motor cortex (in relation to the contiguous cingular cortex [Brodmann’s area 32], located ventral to interhemispheric surface of motor areas), typical large pyramidal neurons of layer V (Betz pyramidal cells) were not detected, showing that different degrees of lissencephalic malformation are heterogeneously distributed in the cortex (Figures 8A,C). A semi quantitative analysis of Nissl-stained sections from the dysplastic frontal cortex (primary motor cortex M1; Figure 8C, right) and the comparison with unaffected cortical areas (Figure 8C, left) showed a homogeneous reduction of neurons (large-medium cell profiles) in lissencephalic superficial cortical layers (2, 3, and 4) and a relative accumulation of neurons in deeper cortical layers (5 and 6). The cortical thickness from layer 2 to layer 6 of these cortical areas were divided in 8 horizontal bands of the same thickness (dotted lines in Figures 8C,D) and the cells in each band were detected and counted with the Image-J software (NIH, United States). Figure 8D shows the neuron counting in the horizontal bands in the lissencephalic area and in the normal cortical area. The analysis of series of parallel sections stained against CB, CR and PV (Figure 8E) showed a clear reduction of GABAergic interneurons (calbindin, calretinin, and parvalbumin positive cells) in upper cortical layers (layes 2 and 3) of the dysplasic (lissencephalic) areas in comparison with not dysplasic (normal) areas (Figure 8F). Our findings related to the distribution of cortical GABAergic interneurons in this case of human lissencephaly were roughly similar to our findings of the altered distribution of GABAergic interneurons in motor cortex of the *Lis1/sLis1* mutant mice; histograms in Figure 2 (mouse data) and Figure 8 (human data) representing CB, CR and PV GABAergic interneurons distribution showed that in both mouse mutant and human motor areas more affected by lissencephaly, CB and CR interneurons showed an inverted distribution, with decreasing number in the superficial cortical layers (2 and 3) and increasing in deep cortical layers (5 and 6); in contrast, PV GABAergic interneurons distribution seems to be more affected in human cortex than in mouse. This may be due to differences among species, with less differential distribution of PV GABAergic interneurons between layers 2-3 and 5-6, even in the normal cortex. However, we cannot establish a direct relationship between this case of human lissencephaly and our model since this animal has a very specific mutation in the *Lis1* gene and is a condition quite far apart from the human case.

## Discussion

In this work, we have reported the presence of abnormalities in the primary motor cortex as a consequence of *Lis1* dysfunction, specifically the LisH deletion in heterozygosity. We showed the presence of morphological as well as functional alterations in the developed cortex of the mutant *Lis1/sLis1* mouse. In the primary motor cortex, we found morphological alterations that were restricted to the distribution of GABAergic neurons and consisted in a relative accumulation of CB, CR and PV positive neurons in deep cortical layers in comparison with superficial layers. The detected functional alterations included in the electrophysiological properties of the FS subtype of cortical interneurons (also including alterations in the sEPSCs) and changes in the electrical cortical activity recorded in anesthetized animals. Overall, our results uncover a number of abnormalities caused by mutation of the *Lis1* gene on the cortical inhibitory system.

### Morphological Abnormalities

It is widely accepted that mutations in the *LIS1* gene cause lissencephaly, a disorder characterized by deep alterations in the organization of the cerebral cortex, ranging from pachygyria to a complete lissencephalic neocortex and including a deep disruption of the laminar organization; these alterations are due to abnormal neuronal migration (Dobyns et al., 1993; Barkovich et al., 2005; Barkovich et al., 2012). In the primary motor cortex of mutant animals, Nissl staining as well as NeuN and layer-specific markers revealed a preserved laminar pattern and layer thickness. Therefore, this model does not display one of the most relevant phenotypical hallmarks of lissencephaly (Dobyns et al., 1993). This cortical laminar pattern is mainly dependent on the arrangement of the pyramidal neurons, which represent around 80% of the cortical neurons (De Felipe and Fariñas, 1992); in consequence, the normal thickness and neuron density of layers 2/3, 5, and 6 indicate that the final outcome of the processes of proliferation and migration of pyramidal neurons is mostly conserved in mutant *Lis1/sLis1* mice. In contrast, the distribution of CR, CB and PV population among superficial and deep layers was clearly disrupted in the mutant mice at postnatal day 30, when interneuron migration (both radial and tangential) was complete (Lim et al., 2018). The relative distribution of CB and CR positive neurons in the superficial and deep layers was inverted in the mutant cortex, presenting more neurons in the deep layers with respect to the superficial ones. This change is very significant, as the distribution pattern of these inhibitory neurons among cortical layers is highly conserved along mammalian phylogeny (Hof et al., 1999). The relative amount of PV positive cells was also significantly decreased in the superficial layers, while increased in the deeper layers. The alteration in the number and distribution of interneurons seems to be a general feature of the *Lis1/sLis1* neocortex, since in the prefontal cortex a lower number of PV and CR positive GABAergic neurons were detected (García-López et al., 2021). Although these neuronal markers (PV, CB, and CR) reveal overlapping populations of GABAergic neurons we explore the total population of cortical GABAergic neurons. CR marks all SST positive neurons and approximately the 40% of the VIP positive and 5HTR3A positive neurons (Tremblay et al., 2016); the remaining 60% of the 5HTR3A (those that are VIP negative) are marked by CB (Cauli et al., 2000).

The clear tendency of the GABAergic interneuron cell bodies’ accumulation in the deeper layers suggests that the radial migration component of the interneuronal migration was affected in the mutant mouse. The arrival of interneurons to the developing cortex and their correct placement within cortical layers during development is critically dependent on both radial and tangential migration (Marin and Müller, 2014). In the *Lis1/sLis1* mutant mouse the radial glia, a key player during radial migration, show an abnormal morphology during prenatal cortical development (Cahana et al., 2001). However, given that pyramidal neuron migration, which depends exclusively on radial migration, was only delayed during prenatal stages (Cahana et al., 2001) in the *Lis1/sLis1* mutant mouse the migratory alterations of interneurons must be a consequence of abnormal tangential migration. In fact, it is already known that interneuron tangential migration is highly susceptible to LIS1 loss- or gain- of function (McManus et al., 2004; Bi et al., 2009). However, it is important to note that we do not have evidences of abnormalities of tangential migration in the *Lis1/sLis1* mouse, and that observations of altered tangential migration as consequence of *Lis1* dysfunction (McManus et al., 2004; Bi et al., 2009) were obtained in mouse models with different *Lis1* mutations than the *Lis1/sLis1* model. Overall, conclude that LisH deletion in heterozygosis specially affects the positioning of GABAergic interneurons but not affect the excitatory glutamatergic neurons in the primary motor cortex, most likely by somehow affecting the tangential migration. Thus, upon this genetic condition, the six-layer laminar pattern in the neocortex is preserved.

Our findings on the distribution of cortical GABAergic interneurons in the *Lis1/sLis1* model are quite similar to those observed in a case of human lissencephaly with a mutation in the lissencephaly critical region (17p13.3) (Figure 8). Interestingly, alterations in these regions have been related with schizophrenia and bipolar disorders (Tabares-Seisdedos et al., 2006; Tabarés-Seisdedos et al., 2008). In this case a partial lissencephaly was present (bilateral fronto-parietal lissencephaly and temporal pachygyria), an abnormality in the distribution of cortical neurons in the frontal lissencephalic area was detected, with a general tendency to the accumulation of GABAergic interneurons in the deep cortical layers. Although a certain degree of lissencephaly is presented in the human case described above, the GABAergic interneurons disruption pattern appears to be clearly altered and is similar to the one described in the *Lis1/sLis1* mutant phenotype, showing that some mutations associated to the *PAFAH1B1* gene can affect specially the cortical inhibitory component.

Although the morphological alterations produced by the deletion in the LisH domain mouse do not fit with the described phenotype of lissencephaly, they are more similar to those described in non-drastic neurodevelopmental disorders, such as schizophrenia, bipolar disorder or autism. For instance, already in 2002, Eyles et al. reviewed a wide variety of alterations in CB, CR and PV GABAergic interneuron distribution and density described in the neocortex of different cohorts of schizophrenic or bipolar patients, while preserving the normal architecture of the brain. Similarly, in autism, there are various reports describing alterations restricted only to GABAergic interneurons density and positioning, especially in parvalbumin positive GABAergic interneurons (Filice et al., 2016)., These phenotypic descriptions are analogous to those described in the *Lis1/sLis1* mutant mice primary motor cortex.

### Oscillatory Electrical Activity Alterations

The electrophysiological properties of cortical neurons as well as their interconnections forming cortical circuits generate the cortical electrical activity; therefore, the presence of altered electrophysiological properties in cortical GABAergic interneurons could result in alterations of the cortical electrical activity. The LFP recordings performed in anesthetized mice indicated that oscillatory electrical activity in the primary cortex *Lis1/sLis1* mutant mouse was altered. Together, functional connectivity synchronizing different neuronal elements of each layer circuit and also connecting different layers are impaired. Thus, oscillatory electrical activity patterns produced is altered and also how the different layers are synchronized, especially at beta and gamma bands. This is functionally relevant as a switch from beta to gamma activity underlie the change from resting state to motor execution in the primary motor cortex (Davis et al., 2013). Subsequently, an abnormal capability of the cortical circuits to get synchronized in both frequency bands could alter the switching between resting state and output execution.

Another important feature of the cortical activity in the *Lis1/sLis1* mutant mouse was the complete absence of spontaneous epileptiform-like activity; the presence of recurrent and serious epileptic seizures, commonly resistant to pharmacological treatment, is a hallmark of typical lissencephaly. In contrast, the increase in power and coherence of oscillatory electrical activity is a hallmark of other less-drastic neurological disorders. Thus, the increased power in oscillatory activity has been described as a characteristic of EEG alteration in schizophrenia and bipolar disorder in humans (Kikuchi et al., 2011; Spencer 2012; Brealy et al., 2014; Liu et al., 2014), as well as in schizophrenia-like animal models (del Pino et al., 2013). Also, an increased interhemispheric and regional intra-hemispheric coherence has been described in schizophrenic patients (Krishna Tikka et al., 2012). This finding of absence of spontaneous epileptiform activity reinforces the *Lis1/sLis1* mouse as a model with non-drastic phenotype, since other commonly used model (the *Lis1* ^*+/−*^ mouse) shows clear spontaneous epileptiform seizures (Greenwood et al., 2009).

### Alterations in Fast Spiking GABAergic Interneurons

A number of functional alterations were detected in the mature cortex of *Lis1/sLis1* mice. The observation of a smaller AP amplitude in layer 2/3 FS neurons, a more negative threshold in layer 6 and a shorter AHP in layers 2/3 and 6 suggest that certain ion channels may be altered. These include the voltage-dependent sodium channels that are responsible for the threshold and depolarizing phase of the AP, as well as the potassium channels implicated in the repolarizing phase of the AP and in the AHP. There is no evidence pointing to any particular type of ion channel, although the underlying alteration could be caused by the possible consequences of *Lis1* mutations on microtubule dynamics, intracellular transport and membrane trafficking, which could affect the transport and turnover of membrane proteins (Lipka et al., 2013); in fact, a delay in the turnover of subunits of the GABA_A_ receptors has been reported in the *Lis1/sLis1* mouse (Valdés-Sanchez et al., 2007). An interesting finding was the increased firing rates observed in layers 2/3 and 6 FS neurons in response to depolarizing current pulses (Figure 7A). FS firing rate has a central role in local network activity synchronization, particularly in the gamma range (Bartos et al., 2007), and abnormal firing as our finding in layers 2/3 and 6, could have consequences upon gamma rhythm properties (González-Burgos and Lewis, 2008). Both layer 2/3 and layer 6 FS neurons from *Lis1/sLis1* animals presented shorter AHP and the amplitude of the AHP was smaller in layer 6 *Lis1/sLis1* neurons; these differences could account for the higher firing frequency observed in these neurons from *Lis1/sLis1* animals. However, in contrast to layer 2/3, the firing frequency in layer 6 *Lis1/sLis1* neurons was clearly higher only up to frequencies of 90 Hz. This difference could be due to compensatory mechanisms, such as homeostatic plasticity, which would maintain the neuronal firing frequency (of both FS and pyramidal neocortical neurons) under different conditions (Turrigiano, 2012; Hengen et al., 2013). It is important to note that the *LIS1* mutation in our *Lis1/sLis1* model is present along the whole process of cortical development and a therefore a maintained lower excitatory input on FS neurons (as shown by the lower frequency of sEPSC) could cause a homeostatic plasticity increase of the firing frequency (shown by the response to depolarizing current pulses) to keep constant the average firing frequency during long periods of time.

The sEPSC recorded in FS neurons from layers 2/3 and 5 (but not layer 6) also presented certain differences in *Lis1/sLis1* animals. Specifically, we detected a lower frequency and an increased peak amplitude (the latter only in layer 2/3 neurons). In layer 2/3 FS neurons we found an increased excitability as suggested by the higher firing frequency (see Figure 7) but they receive a lower tonic excitatory synaptic input (Figure 6); this could be a consequence of homeostatic plasticity, as noted above. It has been shown (Malkin et al., 2014) that in cortical fast spiking GABAergic interneurons recorded in brain slices the mEPSC detected in the presence of tetrodotoxin show the same frequency and the same properties that the sEPSCs recorded before tetrodotoxin; this indicates that the spontaneous excitatory currents recorded in FS neurons were mostly miniature synaptic currents. This suggest that in layer 2/3, both presynaptic (those controlling the frequency) and postsynaptic (those controlling the amplitude) mechanisms are implicated in the changes observed in sEPSCs in mutant animals; in contrast, in layer 5, only presynaptic mechanisms seem to be affected since only the frequency of sEPSC was different in mutant animals. It is interesting that in the hippocampus of another model mouse of *Lis1* dysfunction (the *Lis1* ^*+/−*^ mouse), there are also changes in the interneurons (Jones and Baraban, 2007); in this model, the frequency of sEPSC recorded from regular spiking non-pyramidal neurons was larger in mutant neurons, but their amplitude was similar in mutant and WT neurons. These results are different from our findings in FS neocortical neurons, and these differences may indicate that the consequences of *Lis1* dysfunctions are heterogeneous among brain areas and neuronal types and also that different mutations would have different pathophysiological consequences.

The alterations in synaptic transmission observed in *Lis1/sLis1* animals may have two non-excluding explanations. First, the protein LIS1 is a regulator of diverse transport mechanisms, and its dysfunction could affect the incorporation of neurotransmitter receptors to the postsynaptic terminal. An alteration in the turnover of postsynaptic GABA receptors subunits has been described together with a reduction of mIPSC amplitude in pyramidal neurons of layer 2/3 in the *Lis1/sLis1* mouse (Valdés-Sánchez et al., 2007). Second, LIS1 is needed for the correct establishment of new synaptic contacts among neurons (Kawabata et al., 2012; Sudarov et al., 2013); thus, a LIS1 dysfunction could cause a reduction of excitatory connections established on PV positive interneurons explaining the decrease of the number of sEPSC detected in these neurons.

### LIS1 Dysfunction Beyond the Classical Lissencephalic Phenotype

As we mentioned before, the *Lis1/sLis1* mutant mouse did not recapitulate the morphological and functional lissencephalic phenotype traditionally assigned to a LIS1 dysfunction. Interestingly, the phenotype described is more related to less-drastic neurodevelopmental disorders such as schizophrenia, bipolar disorders, and autism.

Around 30 years ago, the *LIS1* gene was discovered as the gene mutated in the Miller-Dieker syndrome and Lissencephaly type 1 neurological disorders. Since then, some studies have tried to establish a genotype-phenotype relationship among different LIS1 alterations along with the other functional domains in the protein as well as their broader consequences on the lissencephaly. Although a previous study has found no genotype-phenotype relationship in a large cohort of patients, including different mutations (Saillour et al., 2009), LisH mutations have been related with less severe forms of lissencephaly than mutations affecting to other parts of the protein (Kim et al., 2004). The LisH deletion present in the mutant mice from the *Lis1/sLis1* line was designed mimicking a mutation described in a patient with a mild form of lissencephaly (Fogli et al., 1999; Cahana et al., 2001). On the other hand, it has been proposed that LIS1 may be implicated in other brain disorders less drastic than lissencephaly, such as schizophrenia or bipolar disorder (reviewed by Reiner et al., 2006; Bi et al., 2009). This is based on a series of evidence. First, a dysfunction of some LIS1 protein-protein interacting is altered in patients and animal models of these pathologies (Impagnatiello et al., 1998; Guidotti et al., 2000; Millar et al., 2000; Nothwang et al., 2001). Second, the same basic processes such as neural migration or cell polarity establishment, where LIS1 action is necessary, are also affected in schizophrenia or bipolar disorders (Kalus et al., 1997; Reiner et al., 2006; Weigel et al., 2010). Finally, mutations have been reported in the lissencephaly critical region in patients with schizophrenia and bipolar disorders (Tabarés-Seisdedos et al., 2006, 2008). Despite that, there is still no evidence in human patients nor animal models showing that alterations in *LIS1* result in this neurological phenotype. However, mutations in *NDE1*, which is one of the major LIS1-proteins interactors, result in a wide spectrum of brain diseases, including schizophrenia (Bradshaw and Hayashi, 2017). Overall, we described in the neocortex of the animal model *Lis1/sLis1* a series of alterations analogous to different cohorts of patients or animal models of non-drastic neurobiological disorders (García-López et al., 2021).

## Data Availability

The raw data supporting the conclusions of this article will be made available by the authors upon reasonable request.
